# My Bleeding Nephrons!

**DOI:** 10.1177/2324709619858126

**Published:** 2019-06-19

**Authors:** Jiemin Li, Sandeep Anand Padala, George Hinnant, Anusha Vakiti, Azeem Mohammed

**Affiliations:** 1 Medical College of Georgia, Augusta, GA, USA; 2 Augusta University Medical Center, Medical College of Georgia, Augusta, GA, USA; 3 Medstar Washington Hospital Center, Washington, DC, USA

**Keywords:** anticoagulation-related nephropathy, acute kidney injury, warfarin-associated nephropathy, IgA nephropathy, nonalcoholic steatohepatitis

## Abstract

Anticoagulation-related nephropathy (ARN) is an uncommon diagnosis that should be considered in patients presenting with unexplained acute kidney injury (AKI) and coagulopathy. In this article, we present the case of a 70-year-old male with a history of cirrhosis and portal vein thrombosis on Coumadin who presented to the hospital with gross hematuria. The patient was diagnosed with AKI on chronic kidney disease (CKD) secondary to ARN superimposed on sclerosing IgA nephropathy. ARN, also known as warfarin-associated nephropathy, is an uncommon condition in which AKI from glomerular hemorrhage develops in a patient with an international normalized ratio greater than 3. The most common risk factor for development of ARN is CKD. AKI in our patient unearthed preexisting CKD due to IgA nephropathy as evidenced by the biopsy.

## Background

Anticoagulant-related nephropathy (ARN), also referred to as warfarin-related nephropathy (WRN), is a form of acute kidney injury (AKI) caused by excessive anticoagulation. ARN has been associated with irreversible kidney damage and increased risk of mortality. ARN usually occurs during the first 4 weeks of use of anticoagulant therapy and is mostly seen in patients with an international normalized ratio (INR) of 4 and above.^1^ The risk of ARN may be increased in patients with underlying chronic kidney injury (CKD), as several studies have demonstrated increased incidence of ARN in patients with CKD compared with those without it.^2^ Patients with CKD are at increased risk due to reduced renal elimination, increased plasma levels of anticoagulants, and having supratherapeutic INRs despite lower doses.

AKI has also been seen in patients with supratherapeutic INR without any history of underlying kidney disease, thus suggesting heterogeneity in presentation of ARN.^1^ The pathogenesis of ARN involves glomerular hemorrhage, obstruction of renal tubules by red blood cell (RBC) casts, and tubular epithelial cell injury. Excessive anticoagulation leads to glomerular hemorrhage, evidenced by the presence of RBCs in the glomerulus and RBC casts within tubules on histopathology.^1^ In this article, we describe a patient with chronic sclerosing IgA nephropathy who developed WRN following treatment of portal vein thrombosis with warfarin.

## Case Report

### Anticoagulation-Related Nephropathy in a Patient With IgA Nephropathy

A 70-year-old white male with past medical history significant for cirrhosis secondary to nonalcoholic steatohepatitis, portal hypertension status post transjugular intrahepatic portosystemic shunt, portal vein thrombosis on warfarin, hypertension, and hypothyroidism presented to the hospital with chief complaint of hematuria. On admission, his blood pressure was 125/57 mm Hg, heart rate was 55 beats per minute, and temperature was 37.5°C. Laboratory studies demonstrated a blood urea nitrogen/creatinine ratio of 41/4.49 (baseline ~1.38), hemoglobin 10.9 g/dL, white blood cell count 6 K/UL, platelets 120 K/UL, and INR 8.7. Urinalysis was remarkable for too numerous to count RBCs, 20 to 50 white blood cells, large leukocyte esterase, and 3+ protein. A review of the sediment showed numerous RBCs and hyaline casts without any dysmorphic RBCs. Renal ultrasound revealed right kidney measuring 12.2 cm and left kidney measuring 12.6 cm with normal cortical thickness, no evidence of hydronephrosis, and mildly increased echogenicity bilaterally. Given the supratherapeutic INR and ongoing hematuria, warfarin was held and treatment was initiated with intravenous fluids and antibiotics for possible urinary tract infection. Urine cultures were pending.

**Figure 1.**
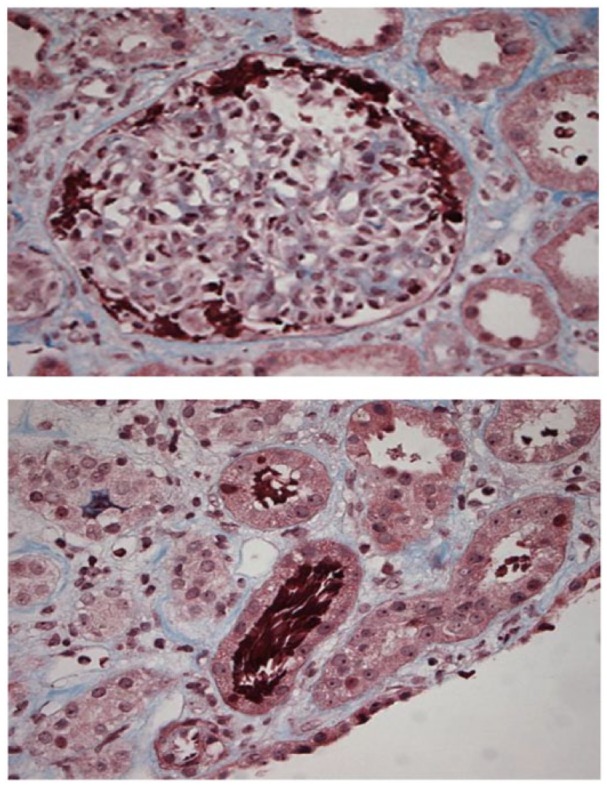
Light microscopy of tubular lumen occlusion by red blood cell (RBC) accumulation within Bowman’s space and tubular RBC casts without necrosis or crescent formation.

Secondary workup for the etiology of AKI revealed 2.8 g of proteinuria on a 24-hour sample and a positive antinuclear antibody at 1:80 dilution. Acute hepatitis panel, double-stranded deoxyribonucleic acid, rapid plasma reagin, rheumatoid factor, complement levels (C3 and C4), and antineutrophil cytoplasmic antibody profile were all negative. Serum protein electrophoresis and serum immunofixation demonstrated a polyclonal IgA. Serum-free light chains ratio was 1:2. Urine cultures were consistent with pan-sensitive *Escherichia coli*. Despite conservative measures, the patient’s renal function continued to worsen with peak creatinine of 8.6 mg/dL, and a renal biopsy was performed. Light microscopy demonstrated sclerotic glomeruli with mesangial expansion and accumulation of red cells within Bowman’s space. Tubular cross section demonstrated RBC casts. There was no necrosis or crescent formation (Figure 1). Electron microscopy (EM) demonstrated scattered mesangial and subendothelial immune complex deposits staining positive for IgA. The patient was diagnosed with ARN superimposed on sclerosing IgA nephropathy. He required 5 sessions of hemodialysis prior to discharge. Later, his creatinine stabilized to 2.5 mg/dL and proteinuria improved from 2.8 g to 1.1 g at a follow-up visit in the clinic.

### Pathology Report (August 23, 2018)

**Figure 2.**
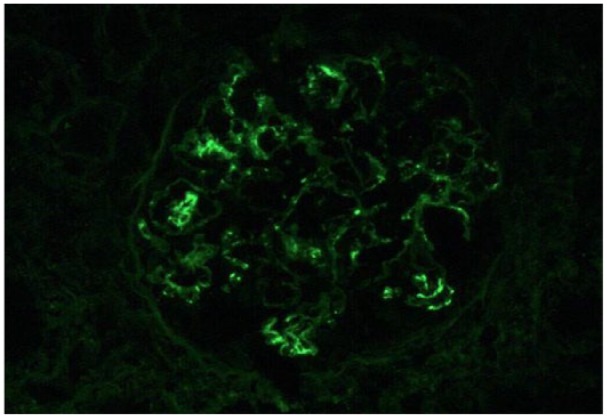
Immunofluorescence demonstrating IgA mesangial and subendothelial immune complex deposits.

Up to 5 glomeruli, 2 of which are globally sclerotic, are identified per level. The glomeruli show mesangial expansion. One glomerulus shows segmental sclerosis. One intact glomerulus shows accumulation of red cells within Bowman’s space. No necrosis or crescent formation is identified. There is mild interstitial fibrosis. Occasional tubular cross sections show red cell casts while other tubules show granular casts. Limited sampling of the vasculature did not reveal any abnormalities. The biopsy examined via immunofluorescence consists of 2 tissue fragments containing renal cortex. Up to 19 glomeruli with no globally sclerotic glomeruli are identified per level. IgA and C3 are identified within the glomeruli as listed below. By immunofluorescence, the immune complexes appear to be mesangial and along the capillary loops. Ultrastructural evaluation reveals scattered mesangial and occasional subendothelial immune complex-type dense deposits (Figure 2).

### EM Report (August 23, 2018)

The glomeruli show mesangial expansion with segmental sclerosis. Ultrastructural evaluation reveals scattered mesangial immune complex-type dense deposits and mesangial expansion. There are occasional subendothelial immune complex-type dense deposits. The podocytes are hypertrophic with diffuse foot process effacement.

## Discussion

ARN should be suspected in the setting of use of anticoagulation, hematuria, and unexplained AKI. It is defined as AKI without any obvious underlying cause, in a patient with an INR >3.0 and microscopic or gross hematuria. Though previously thought to be caused only by warfarin (and thus referred to warfarin-induced nephropathy), ARN may also be caused by new oral anticoagulants such as dabigatran.^3^ Our report describes a case of ARN in the setting of chronic sclerosing IgA nephropathy.

The risk of WRN is higher in patients with underlying CKD, multiple risk factors for AKI, and other comorbidities. History of CKD is the strongest risk factor. Several mechanisms of ARN-induced AKI have been proposed, including tubular luminal obstruction by RBC casts; direct toxicity to tubules from release of hemoglobin, heme, and iron; and ischemic and inflammatory changes leading to tubule destruction.^4^ Though ARN is a serious condition that significantly increases patient morbidity and mortality, it remains underdiagnosed for several reasons. Until recently, ARN was thought to result only from severe degree of coagulopathy; however, several cases reported by Brodsky et al suggested that milder degree of coagulopathy could also result in AKI.^5^ ARN is associated with significant morbidity and mortality, causing it to be underrepresented in those treated with anticoagulation. Additionally, due to procedural risk of bleeding, practitioners are hesitant to perform renal biopsies to confirm the diagnosis.

To our knowledge, only 4 cases of ARN in patients with IgA nephropathy have been reported, of which 2 patients were treated with dabigatran,^3^ one patient was treated with acenocoumarol,^6^ and only one patient was treated with warfarin.^7^ IgA nephropathy leads to glomerular hemorrhage and macroscopic hematuria through RBC accumulation within the glomerulus and casts that can occlude the tubules. Subsequent events including interstitial hemorrhage and oxidative stress on the kidney lead to the overall progression of ARN.^4^ Thrombin, which stimulates coagulation via activated vitamin K, binds and activates a series of proteinase-activated receptors, located in tubular endothelial cells, increasing endothelial permeability.^8^ Anticoagulant use leads to a decrease in thrombin and subsequent disruption of this endothelial layer, increasing permeability and leading to glomerular hemorrhage. Protein C, which is involved in the coagulation cascade, has also been recently shown to be involved in podocyte integrity.^8-12^ Disruption of protein C signaling by anticoagulants may be another potential factor in the pathogenesis of ARN.

Risk factors in our patient included undiagnosed IgA nephropathy, hypertension, coronary artery disease, and heart failure. Renal function was normal prior to the episode, with creatinine of 1.38 mg/dL. However, biopsy findings demonstrated that there were several indicators of chronic kidney damage, including mild interstitial fibrosis and segmental sclerosis; however, no vascular abnormalities such as arterial intimal thickness were found. Biopsy also revealed increased mesangial thickness. These findings suggest that despite a normal baseline creatinine, the patient might have had underlying CKD, thus predisposing him to ARN. In addition, EM findings demonstrated podocyte effacement, consistent with the hypothesis that compromise of podocyte integrity from anticoagulant use may contribute to the pathogenesis of ARN.^9^

Treatment for ARN is largely supportive and involves adjusting the patient’s medications to reach a therapeutic INR. Warfarin was held for our patient until the INR stabilized from 8.7 to 3.3. In addition, the patient’s creatinine gradually improved and stabilized approximately 2 months after the initial insult. There are limited options for treating ARN, and thus ideal management involves early detection and prevention of the disease. However, this is complicated by the fact that ARN remains a diagnosis of exclusion unless patients receive a renal biopsy, which is infrequently performed due to the increased risk of bleeding. Any patient on anticoagulation with worsening renal function should be evaluated for the possibility of ARN. In addition, patients should have their INR monitored regularly on initiation of warfarin, and dose adjustments or cessation should be done in the event of laboratory disturbances. Further research regarding the pathogenesis, risk factors, and treatment of ARN would help diagnose this rare disorder better and provide more insight into its management. The role of steroids is unclear, but it was used for management in a reported case.^13^

## References

[1] Brodsky SV Nadasdy T Rovin BH et al Warfarin-related nephropathy occurs in patients with and without chronic kidney disease and is associated with an increased mortality rate. Kidney Int. 2011;80:181-189. 21389969 10.1038/ki.2011.44 PMC3675881

[2] Limdi NA Beasley TM Baird MF et al Kidney function influences warfarin responsiveness and hemorrhagic complications. J Am Soc Nephrol. 2009;20:912-921. 19225037 10.1681/ASN.2008070802 PMC2663833

[3] Escoli R Santos P Andrade S Carvalho F Dabigatran-related nephropathy in a patient with undiagnosed IgA nephropathy. Case Rep Nephrol. 2015;2015:298261. 26347498 10.1155/2015/298261 PMC4540981

[4] Cleary CM Moreno JA Fernández B et al Glomerular haematuria, renal interstitial haemorrhage and acute kidney injury. Nephrol Dial Transplant. 2010;25:4103-4106. 20709744 10.1093/ndt/gfq493

[5] Brodsky SV Satoskar A Chen J et al Acute kidney injury during warfarin therapy associated with obstructive tubular red blood cell casts: a report of 9 cases. Am J Kidney Dis. 2009;54:1121-1126. 19577348 10.1053/j.ajkd.2009.04.024

[6] Ng CY Tan CS Chin CT et al Warfarin related nephropathy: a case report and review of literature. BMC Nephrol. 2016;17:15. 26830352 10.1186/s12882-016-0228-4 PMC4736492

[7] Góis M Azevedo A Carvalho F Nolasco F Anticoagulant-related nephropathy in a patient with IgA nephropathy. BMJ Case Rep. 2017;2017:bcr2016218748. 10.1136/bcr-2016-218748 PMC5318584 28219912

[8] Coughlin SR Thrombin signalling and protease-activated receptors. Nature. 2000;407:258-264. 11001069 10.1038/35025229

[9] Madhusudhan T Wang H Straub BK et al Cytoprotective signaling by activated protein C requires protease-activated receptor-3 in podocytes. Blood. 2012;119:874-883. 22117049 10.1182/blood-2011-07-365973 PMC3398751

[10] Kabir A Nadasdy T Nadasdy G Hebert LA An unusual cause of gross hematuria and transient ARF in an SLE patient with warfarin coagulopathy. Am J Kidney Dis. 2004;43:757-760. 15042555 10.1053/j.ajkd.2003.08.050

[11] Nieman MT Protease-activated receptors in hemostasis. Blood. 2016;128:169-177. 27127302 10.1182/blood-2015-11-636472 PMC4946198

[12] Teng B Duong M Tossidou I Yu X Schiffer M Role of protein kinase C in podocytes and development of glomerular damage in diabetic nephropathy. Front Endocrinol (Lausanne). 2014;5:179. doi:10.3389/fendo.2014.00179 25414693 PMC4220730

[13] Golla A Goli R Nagalla VK Kiran BV Raju DSB Uppin MS Warfarin-related nephropathy. Indian J Nephrol. 2018;28:378-381. doi:10.4103/ijn.IJN_3_17 30271000 PMC6146722

